# Informal Caregivers’ Health Literacy in Lisbon, Portugal: A Profile for Health Promotion Prioritization

**DOI:** 10.3390/geriatrics7050092

**Published:** 2022-09-06

**Authors:** Andreia Costa, David de Sousa Loura, Paulo Nogueira, Graça Melo, Idalina Gomes, Isabel Ferraz, Laura Viegas, Maria Adriana Henriques

**Affiliations:** 1 Nursing Research, Innovation and Development Centre of Lisbon (CIDNUR), Nursing School of Lisbon (ESEL), 1600-096 Lisbon, Portugal; 2 Instituto de Saúde Ambiental (ISAMB), Faculdade de Medicina, Universidade de Lisboa, 1649-028 Lisbon, Portugal; 3 Nursing School of Lisbon, 1600-096 Lisbon, Portugal; 4 Hospital Dona Estefânia, Centro Hospitalar Universitário de Lisboa Central, E.P.E., 1169-045 Lisbon, Portugal; 5 Laboratório de Biomatemática, Instituto de Medicina Preventiva e Saúde Pública, Faculdade de Medicina, Universidade de Lisboa, Avenida Egas Moniz, 1649-028 Lisbon, Portugal

**Keywords:** health literacy, informal caregivers, nursing, survey, community

## Abstract

Health literacy (HL) allows people to access, understand and evaluate health information. Informal caregivers’ levels of HL may impact long-term care outcomes. ‘Informal caregivers’ profile in Lisbon county: a health community approach’ is a nurse-led research project aiming to assess informal caregivers’ health literacy and associated factors in Lisbon county, as well as to foster the development of a local-specific health literacy strategy. A survey to identify a health/social caregiver profile, including questions about HL (HLS-EU-PT), was submitted to a representative sample of carers. Descriptive and bivariate inferential analysis was developed. Informal caregivers’ level of HL was mostly sufficient (*n* = 99, 38%). More than 60% of caregivers have limited HL regarding health promotion. ‘Access’, ‘Appraisal’ and ‘Use’ are the information processing stages with lower mean scores of HL. Carers with low HL levels appear to be older and to have less education, low knowledge of community resources and decreased wellbeing (*p* < 0.05). A strategy focused on health promotion-related HL through primary care resources can potentially improve caregivers’ knowledge, competencies and motivation, as well as health system sustainability. Reported HLS-EU-PT scores deserve special attention. Future work should emphasize the role of HL-associated factors and health outcomes for caregivers and cared-for persons.

## 1. Introduction

The contrast between advances in science and technology and a strong increase in the prevalence of non-communicable diseases (NCDs) reveals that our population lives for a longer time but with fewer years free of disease. Published data show that between 1990 and 2016, healthy life expectancy was set between 68.7 and 72.3 years old [1], as the birth rate decreased around three percent in the same period [2]. However, the same data reveal a parallel rising trend in the years lived with dependence, particularly regarding non-communicable diseases [1].

Therefore, a strategy designed to care for people with chronic diseases is becoming more and more necessary, particularly following the trajectory of the disease and supporting compliance with the therapeutic regimen. Long-term care is hereby essential to ensure the quality of life for these people, which remains a challenge in the European Union since there is still a significant portion of care demand that is not being met [3] and inconsistencies between long-term care systems across Europe [4]. 

Thus, long-term care relies mostly on the work of informal or family caregivers, compensating for the lack of formal long-term care services and/or the difficult access to these facilities [5]. According to Eurocarers, there were approximately 71 million caregivers in the EU in 2021 [6]. In Portugal, around 12.5% of the population was identified as caregivers. The growth of informal carers is, therefore, undeniable. At an economic level, the work of informal caregivers is equivalent to at least 2.5% of European GDP, spending more than 33 billion hours per year on this activity [4].

OECD defines informal caregivers as someone who provides any type of help to the members of the family, friends and social network in need for support with daily activities [7]. The activity of a caregiver involves tasks such as helping with basic and instrumental activities of daily living (such as bathing, dressing, shopping, and housework, among others) and emotional support, also including a role of management and coordination with the necessary resources to ensure the quality of care to the cared-for person [4]. 

Some authors state that caring for one another is a unique experience of interaction between the personal characteristics of the caregiver and the cared-for person, in which there is a lot of tension, effort and complex tasks which are difficult to deal with and manage [8,9]. This context can be particularly challenging when the caregiver must manage the therapeutic regimen of a cared-for person with an NCD, involving multi-skilled tasks such as knowledge about the disease, the ability of managing signs and symptoms and the adoption of health promoting behaviours, which they are not always ready to respond to [10]. 

Educating caregivers, and promoting the acquisition of knowledge and therapeutic regimen management’ competencies, is therefore crucial to ensure positive outcomes of long-term care. In this process, the concept of health literacy cannot be disregarded, as it is in the centre of the 2030 agenda for health promotion (Declaration of Shanghai) as a structural sector to preserve the achievement of sustainable development goals, “empowering individual citizens to demand rights and quality services, and (...) [enabling] engagement in collective health promotion action” [11].

Health Literacy (HL) is defined by the European Health Literacy Consortium as a concept that is connected to people’s knowledge, competencies and motivation to access, understand, and evaluate and apply health information, allowing them to take decisions about healthcare, health promotion and disease prevention that maintain or increase quality of life [12]. 

Investing in HL promotion initiatives is essential. Evidence shows that low levels of HL are associated with an increase in hospital admissions, search and use of emergency services and a decrease in concerns with preventing disease and promoting health-seeking behaviours. These factors result in a declining quality of life [13]. Therefore, compromise with HL promotion and dissemination is key to provide all people with essential skills to deal with their lifestyle and medical conditions, such as critical thinking and information management [14]. In Portugal, a National Strategy for Health Literacy was developed by the Directorate–General of Health (DGS), involving patients, health professionals and stakeholders in the promotion of sound evidence-based professional practice and the investment in promoting people’s health literacy [14].

Measuring health literacy in the population is, therefore, a major challenge. Still, simultaneously, said challenge is a crucial starting point to design targeted health policy, as it allows for the identification of specific groups or areas to improve, ensuring support for understanding, assessing or applying health-related information [15]. Informal caregivers, as vital players providing long-term care, are one of the groups in which there is a special interest in HL measurement since the development of HL skills may impact millions of people worldwide.

Literature on this topic is growing, but it follows a clear trajectory. The latest studies found that low levels of HL were associated with less concerned self-management behaviours of the cared-for person, increased resort to health services and increased caregiver burden [16]. Researchers found that for a caregiver with low HL, the odds of finding health communication and system navigation harder were 2.52 times higher than for a carer with an adequate level of HL [17]. More recently, a study from Norway also identified significant associations between high values of HL and a decrease in caregiver burden and time spent on informal care, as well as an increase in health-related quality of life [18].

Promoting HL of caregivers is, therefore, an important step to ensure their education and, therefore, a way to reduce the burden of the disease in the caring activity to guarantee proper support, enhance active ageing and the maximum preservation of autonomy [5]. ‘Informal caregivers’ profile in Lisbon county: a health community approach’ is a nurse-led research project designed to characterize informal or family carers in Lisbon county, considering social and demographic parameters, along with their needs and perception on their role. HL assessment and measurement were carried out. Our aim is to (1) assess informal caregivers’ health literacy and associated factors in Lisbon county and (2) foster the development of a health literacy strategy tailored to Lisbon county caregivers’ needs.

## 2. Materials and Methods

The project hosting this study follows a multistudy and multimethod approach [19], and it is being developed through two stages. We report to the first stage, a quantitative study with a descriptive cross-sectional design.

This study gathered a convenient, network-based, and stratified sample [20], which resulted from applying inclusion and exclusion criteria, as described in Table 1. The identification of eligible participants for the study was developed in close collaboration with 33 institutional partners, such as non-governmental organizations, municipality, and community healthcare structures. These institutions were the basis for the stratification of the sample. 

A data collection survey was developed, including sections about the cared-for person, information about the caregiver and evaluation scales about the caregiver. Questions about sociodemographic and family information, as well as on caregiving facilities’ conditions, social status, and health history, were included. Prior to survey administration, an internal review of the data collection survey was conducted, gathering the expertise of the research team in identifying dubious questions and inaccuracies that could hamper the form filling, as well as performing internal pilot testing of the questionnaire. 

Caregiver evaluation scales regarding two topics were included (in the Portuguese version) with proper authorization by the authors: Health Literacy (HLS-EU-PT) [21,22]; Well-Being (Well Being Index—WBI-WHO-5) [23].

HLS-EU-PT is an instrument that identifies awareness of basic competencies, such as knowledge, information, cognitive and social skills, lifestyle, attitudes and values, motivation, and medical management [24]. The assessment of health literacy is conducted through 47 questions categorized in three dimensions—healthcare, disease prevention, and health promotion—and is divided by four levels of information processing—access, understanding, evaluation, and information assessment—designed to measure the self-perceived difficulty of health-related relevant tasks [25]. These 47 items were assessed using a 4-point self-report scale (very easy, easy, difficult, very difficult). Therefore, the HLS-EU measures self-perceived HL and reflects about the way that individual competencies can respond to situational complexities or demands.

Each group of questions originated an index, which was calculated through a unified metric from 0 to 50 points, where 0 represents the lower level and 50 stands for the higher level of health literacy [25]. The instrument is validated for the Portuguese population, and its psychometric properties are similar to those found in other countries using HLS-EU, presenting a satisfying cronbach alpha coefficient (α = 0.96 for the general index, and 0.90 ≤ α ≥ 0.96 for the specific indexes) and inexistence of redundancy between indexes (Pearson correlation coefficient ≤ 0.85 between all indexes) [26].

Caregivers’ participation in this study was open between March and September 2021, following a structured approach. First, a contact from an institutional partner designed to give a brief context on the project was received by the caregiver. If the dyad was available to participate, a project collaborator would contact the caregiver and the cared-for person to give an extended explanation about their involvement in the study as well as to define its modality: online (through Limesurvey® platform, in a real-time interview or by autonomous response) or on paper (a physical interview conducted in a home visit setting by primary health care nurses). The cared-for person was encouraged to respond to the questions about their sociodemographic and health data when such was possible. All the information on each participant process was registered in an encrypted document, only accessible to the research team and interviewers. 

Regarding statistical analysis, data were archived in the Limesurvey®’ backoffice and exported to Microsoft Excel® and IBM® SPSS® Statistics files. Descriptive univariate analysis was conducted on all variables. We report the descriptive univariate analysis on sociodemographic variables and HLS-EU-PT score/levels, as well as the bivariate statistical analysis developed between HLS-EU-PT score/levels in relation to the other variables, using association (Pearson’s Chi-Square or Fisher’s Exact test, using Monte Carlo simulation when necessary), correlation (Pearson’s r or Spearman’ ρ) and distribution comparisons (*t*-test, ANOVA, Mann–Whitney or Kruskal–Wallis) with parametric and non-parametric tests, as applicable. A significance level (α) of 5% was considered for the results interpretation. 

The research team was composed of a higher nursing school panel of researchers and two nurses as research collaborators. Sixteen undergraduate students in Nursing and nine postgraduate students in Community Nursing and Medical and Surgical Geriatric Nursing collaborated as interviewers. Periodic meetings took place to prepare the study documentation and the survey administration, as well as to connect the team about the ongoing activities. All research team members shared interview guidelines and project’s activities flowchart.

The project obtained a declaration of approval in February 2021, prior to data collection launching, by the Health of Lisbon and Tejo Valley Regional Health Administration’s Ethics Committee (process number 105/CES/INV/2020). All ethical procedures were completed. A free, prior, and informed consent for both the caregiver and the care receiver (due to their joint participation in fulfilling the questionnaire), fully explaining the participation regimen, was signed before survey administration. The information provided by data collection was anonymised and stored in a confidential location. All the researchers and collaborators involved filled in a confidentiality agreement. 

## 3. Results

This study gathered 639 caregivers indicated by partners. About 28% did not participate in the study due to: lack of answer to the phone call (21%); unavailability/no interest in participation (6%); no identification as a caregiver, institutionalization of the cared-for person, and lack of health conditions to participate (1%). These caregivers were not accounted for in the final sample.

The final sample included 460 caregivers, in addition to 71 caregivers who had access to the questionnaire from other platforms. We report a 64% response rate, as 343 caregivers have completed the questionnaire or all the responses until the caregiver information section. In the majority, a caregiver was responsible for providing care to one person, although 11% identified their responsibility for caring for more than one person.

Cared-for persons included in this study were mostly women (72%), aged between 2 and 102 years old (*M* = 80.3; *SD* = 17.2), widowed (45%), and the majority had concluded primary education (40%). Eight out of ten cared-for persons had been provided care since one year ago, and half received care for over six or more years. Disease was identified as the main causal factor of limitation by 56% of the participants, mainly mental and behavioural disorders (22%), followed by diseases of the circulatory system (20%) and diseases of the nervous system (19%). Seven out of ten cared-for persons identified comorbidities.

Regarding cared-for persons’ level of dependence, results from the application of the Barthel Index for Activities of Daily Living [27,28] and the Lawton & Brody Instrumental Activities of Daily Living Scale [29,30] show that these persons have a high dependence profile, with one-third assuming total dependency on basic activities of daily living and more than two-thirds showing total dependence in instrumental activities of daily living.

Concerning informal or family caregivers, results originated a typical profile (Table 2) where their majority is a married (57%) woman (83%), aged between 22 and 94 years old (*M* = 62.3; *SD* = 13.1). Almost half of these caregivers completed at least one higher education level (44%). A big portion of the participants is retired (43%)—18% of those retired to care for the care recipient—and employed caregivers work in several fields, most commonly in intellectual and scientific activities (29%). Approximately four out of ten caregivers received a salary between EUR 665 and 1270 per month. Sociodemographic data about informal caregivers are presented in Appendix A in a more detailed version.

**Figure 1:**
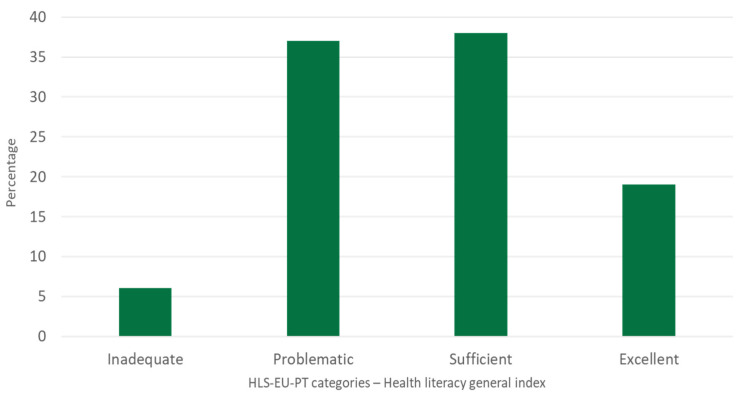
Caregivers’ Health Literacy distribution—Health literacy general index (HLS-EU-PT).

Regarding health literacy, following HLS-EU-PT parameters, caregivers present a 35-point mean score (*SD* = 7.4) of general health literacy, identified as ‘Sufficient’ for 38% of the sample. As Figure 1 shows, although most of the caregivers have an adequate (‘Sufficient’ or ‘Excellent’) level of health literacy, approximately four out of ten carers have a score compatible with limited health literacy (‘Insufficient’ or ‘Problematic’).

**Figure 2:**
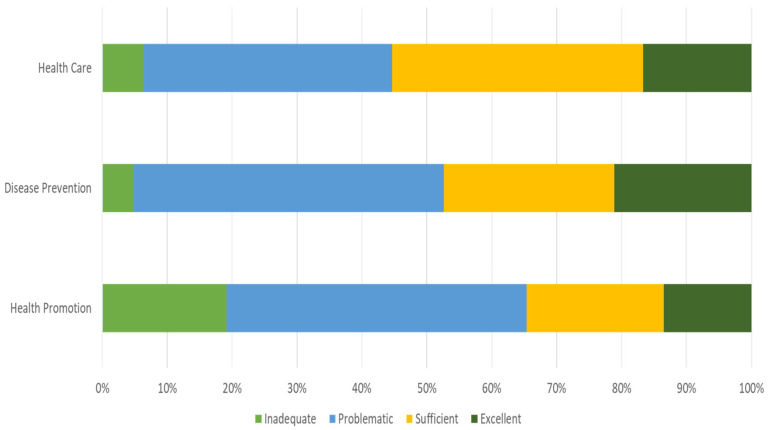
Caregivers’ health literacy distribution by health relevant area/categories—health literacy indexes (HLS-EU-PT).

Dividing health literacy by health-relevant areas, indexes are different among those: for healthcare literacy and disease prevention, a 36-point mean score (‘Sufficient’) is higher than the average score for health promotion, which is 32 points (“Problematic”). These data are corroborated by the health literacy level’s distribution, where scores compatible with limited health literacy for health promotion are observed in more than 60% of the sample, as shown in Figure 2. 

**Figure 3:**
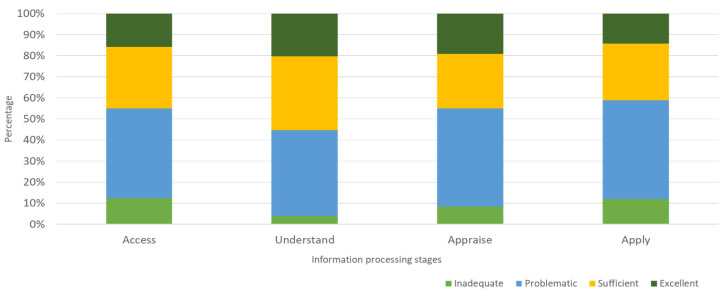
Caregivers’ health literacy distribution by information processing stages/categories (HLS-EU-PT).

In what relates to information processing stages, more than 50% of the caregivers have scores compatible with limited health literacy for access, appraisal and use, as shown in Table 3. ‘Understanding’ appears to be the stage with higher scores of health literacy, since almost 60% of the sample presents ‘Sufficient’ or ‘Excellent’ categories. Figure 3 illustrates health literacy categories’ relative frequencies related to information processing stages. 

Concerning bivariate statistics, health literacy categories and scores were matched with several sociodemographic variables, aiming to explore meaningful inferential connections. To this aim, the health literacy general score and categories were the only considered indexes. Results are synthetically described in Table 4, where the best significance value is reported for each covariate. A full version of this table is available in Appendix B.

Inferential statistics show a highly significant relation between health literacy and gender, knowledge about community resources, and WHO Well-Being Index. A significant relationship between health literacy and age is also described.

**Figure 4:**
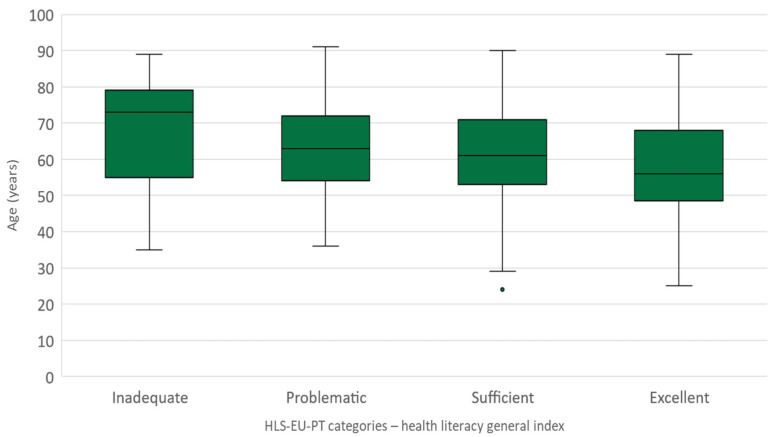
Caregivers’ age distribution by health literacy categories (HLS-EU-PT).

Age distribution through the several categories of health literacy appears to follow a descendent trend when associated with higher levels of health literacy, evidencing lower median values but higher interquartile ranges (Figure 4).

Concerning education, results confirm an association between at least one health literacy category and caregivers’ education level. According to the data shown in Table 5, Higher levels of education, particularly university-level degrees, appear to be linked to more adequate health literacy categories in the HLS-EU-PT instrument. Lack of primary education seems linked to less adequate health literacy categories.

Following these study data, there is also evidence that health literacy levels differ between caregivers with or without knowledge about community resources. Higher scores in the HLS-EU-PT appear to be associated with those who show knowing these resources. A similar relation is reported on the WHO Well-Being Index, where higher scores in this index are significantly correlated with adequate levels of health literacy.

## 4. Discussion

Results described in the previous chapter answer the aims of the article, as an assessment of health literacy in Lisbon County across informal or family caregivers is reported, along with several factors which can foster the development of a specific health literacy strategy in this area.

Literacy has become one of the most central concepts when talking about global health in the last few years. Several documents and resolutions highlight the role of health literacy in sustaining the achievement of individual, local and global goals. Particularly at a national level, the Portuguese’ National Directorate–General of Health (DGS) is implementing a health literacy national plan, designed to promote the adoption of healthy lifestyles, enhance proper use of the health system, improve well-being in people with chronic diseases, and foster knowledge and research [31]. Therefore, results of this study have the potential to raise awareness about this theme and contribute to reaching this plan’s goals positively.

To the best of the authors’ knowledge, the present study is the first to create a local profile on health literacy of Portuguese’ informal caregivers, despite the other studies on general profiles [32,33].

In what concerns the person in need of care profile, results are similar to what is reported in other studies. Particularly for sex, identifying women as the most common care recipient [3,32]; age on average is 80 years old [34]; and the level of dependence, in which a severe classification was the most described status for basic and instrumental daily living activities. Caregiver sociodemographic profile was predominantly overlapped with the conclusions of available scientific evidence on the topic, with slight differences on the financial situation [35,36]. 

Concerning informal caregivers’ health literacy, articulation with scientific evidence is mixed. In this study, the 35-point mean score of HLS-EU-PT general health literacy index, classified as ‘Sufficient’ for 38% of the carers, is above the same index mean score for the Portuguese population calculated in 2016, when the first mapping exercise in Portugal was conducted (*M* = 33.0) [22]. Limited health literacy also seems to have positively developed since 2016, when a 61% rate of this indicator was described [26]. In the period between 2016 and 2022, the Portuguese National Directorate–General of Health created a division designed to tackle health literacy, as well as a specific national-level plan to promote it, which could have led to this increase in HLS-EU-PT general health literacy index score. 

The latest evidence for informal caregivers states a range between 0% and 52.5% of limited health literacy [16], which is compatible with our findings. Concerning health relevant areas and information processing stages, this study reports a lack of recent evidence using HLS-EU as a central informal caregivers health literacy measure instrument. However, the more recent data from 2016 report lower values of HL health care index [26], and higher values of HL disease prevention index and HL health promotion index [22], compared to those estimated in this study. 

Particularly in the case of the HL health promotion index, limited health literacy appears to continue on an ascending trajectory. Several factors can explain this phenomenon, starting with the lack of programs designed to support and educate informal carers in their caregiving role, but also not forgetting about the need for investment in caregiver support networks that promote contact and follow-up by health professionals [37].

Regarding information processing stages, adequate levels of health literacy are only reported for the ‘Comprehension’ dimension. Despite that, adapting caregivers’ educational materials continues to be important, since its language is often targeted at a highly literate population [38].

Several studies corroborate the limited health literacy found in more than 50% of caregivers concerning ‘Access’, ‘Appraisal’, and ‘Application’. Current data from OECD describes that European individuals have significant difficulties accessing and appraising health information from those published in the media [37]. A scoping review involving caregivers of people with cancer also stated that the digitalization of health could be identified as a barrier to health literacy development, mainly to individuals with low socioeconomic status and educational level, as well as reduced familiarity with technology [39].

Furthermore, a caregiver training program in Italy reported a significant impact on caregivers, with improvements in the ability to look for and find health information. Still, it also requires training and emotional/social support [40]. In Portugal, the previously reported lack of caregiver support programs can also lead to limited health literacy in accessing, appraising and applying health information, negatively contributing to raising health literacy levels. This suggestion is supported by a trend found in our study, where 50% of the caregivers that were not involved in support or training programs had HL general index scores below 33 points; meanwhile, 50% of those who were had scores above 38 points (despite that the difference was not statistically significant).

Concerning statistical associations between HL and sociodemographic/professional factors, relations assessed in this study found similar conclusions in the literature. Regarding age and education, in the geriatric and less educated population, Portugal sees a higher risk of presenting a low health literacy general index [22,37]. Furthermore, a study designed to measure HL levels of parents/guardians of paediatric surgery patients found that high scores of HL were positively associated with the educational level of the parent (*p* <0.01) [41]. Since Lisbon has a growing ageing rate and only about 37% of the population goes higher [42], the majority of the caregiving population, which is, on average, aged 80 years old, may not have the resources to increase their health literacy.

Knowledge about community resources is described in this study as a positively associated factor of HL, which is in line with the more recent literature on the topic. A study designed to meet the role of health literacy as a predictor of healthcare system navigation discovered that low levels of caregiver HL is associated with difficulty navigating the healthcare system [17]. Since poor knowledge about community resources may undermine caregivers’ capability to seek support in that context, low HL appears to be an urgent factor to work on, empowering people with knowledge and skills to avoid overloading the health system. This suggestion is in line with a systematic scoping review which concluded that low caregiver HL is associated with increased care recipient use of health services [16].

Evidence regarding the relationship between well-being and HL seems to be an available heuristic area. However, the conclusions of this study are corroborated by a study from China, where caregivers’ knowledge and skills of people with schizophrenia were identified as a significant predictor of well-being [43]. However, a systematic review identified mixed findings associating internet-based support interventions for caregivers to positive outcomes on well-being for most of the sample, despite six of the studies included in the review reporting negative outcomes [44]. The mean value of WHO Well-Being Index for our study, 51 points (*SD* = 24.1), is therefore compatible with a sufficient health literacy level for most caregivers.

Health literacy is a multidimensional concept; therefore, its need for optimization and enhancement must be versatile and multipurpose. This study states the need to raise health literacy of informal caregivers to empower them to provide better care with the best quality of life possible. The literature has shown us that literate people are most suitable to having a more positive interaction with the health system, contributing to long-term care success and, in the long run, a healthier and better-informed population. For that matter, a strategy linked to their knowledge, competencies and motivation is crucial [25], primarily focused in health promotion-related HL through primary health care resources.

Most caregivers presenting limited health literacy concerning accessibility, appraisal, and use of health information should form the basis of a strategy tailored to the needs of Lisbon county, which are unique and linked to a specific social and cultural context. Programs such as awareness campaigns about health literacy or health/disease-related concepts, initiatives focused on evaluating information reliability about a healthy lifestyle, and dynamics that foster motivation to be involved in activities that improve health and well-being can be starting points. Initiatives targeting healthcare and disease prevention-related health literacy are also necessary to positively impact caregivers’ in their role. 

Linking these initiatives not only with the needs of the carer, but also with the profile of the cared-for person, must be pivotal. In this case, adapting approaches to a simple language, since the majority of the people only have a primary education, and target interventions to the areas in which most people manifest a limitation, such as mental, behavioural, circulatory and nervous system diseases, can amplify the impact of HL improvement programs.

The operationalization of these HL-promoting programs can undertake a key role in empowering caregivers. Linking these carers to a supportive community network, as well as adopting healthier lifestyles and behaviours and improving navigation in the healthcare system, has the potential to optimize health services use and associated costs and to improve their well-being.

The limitations of this study are mainly associated with its typology. As a survey-based study predominantly conducted through a digital platform, the lack of personal contact with the caregivers made it hard to constantly connect with them, which in some cases resulted in non-inclusion or incomplete answers. Some participants said they would have preferred to be interviewed with a more personal approach. 

The need for research in this field also configures a limitation since the inferential statistics process found little validation in the literature, mainly concerning knowledge about community resources and well-being. Our sample was network-based, and that may undermine generalizations about the Lisbon caregivers’ population. Nevertheless, it succeeds in finding caregivers and providing their characteristics and some of their information.

## 5. Conclusions

This study provides a local profile of informal caregivers in Lisbon, as well as on their health literacy characterization. The Lisbon informal caregiver is mostly a woman (82%), married (47%), aged on average 62.3 years old (*SD* = 13.1), and in a great percentage retired (43%). The Lisbon caregiver takes care of one person with dependence (89%) and 11% take care of more than one person. The Lisbon informal caregiver shows an interesting mean level of health literacy of 35 points—slightly below the 38% of “sufficient” cut-off—(*SD* = 7.2), with a proportion slightly above 50% with adequate health literacy. 

Enhancing health literacy through the involvement of the caregivers in initiatives aiming to improve access, understanding, appraisal and use of health information can have meaningful outcomes to their well-being and, therefore, to the quality of life of themselves and the cared-for people. Investment in optimizing HL-related healthcare, disease prevention and health promotion systems is a visionary strategy aiming to ensure sustainability and active citizen participation around the globe. Future research is needed to clarify relations among HL, sociodemographic factors and other interest phenomena, and to explore health professionals’ effective interventions on HL promotion in the caregiving domain.

## Figures and Tables

**Table 1:** Inclusion and exclusion criteria.

Inclusion Criteria	Exclusion Criteria
Informal caregiver aged 18 years old or above; Informal caregiver assuming care to a care receiver living in Lisbon county, Portugal; Informal caregiver assuming care to a person with care recipient in a home setting; Informal caregiver assuming care of a person with limitation in at least one dimension of self-care.	Informal caregiver aged 17 years old or below; Informal caregiver living outside the Lisbon county or in other country; Informal caregiver assuming care to a person with dependence in community settings or residential facilities; Formal caregiver assuming care to a person in need; Informal caregiver assuming care to a child without a chronic or disability condition. Person assuming care to another person without limitations of self-care or daily living activities.

**Table 2:** Typical profile of the informal caregiver.

According to This Study, the Informal Caregiver is Most Frequently
a woman aged, in average, 62 years old, married, with a university-level degree, who is retired and earns a monthly financial income between 665 and 1270 euros.

**Table 3:** Characterization of Caregivers’ Health Literacy distribution by information processing stages.

Health Literacy (HLS-EU-PT) Scores by Information Processing Stages	Statistics		
	Mean	Standard Deviation	Median
Access	33.7	8.4	33.3
Understanding	36.6	7.3	34.9
Appraisal	34.7	8.0	33.3
Use	33.3	8.1	33.3

**Table 4:** Bivariate statistics—health literacy and sociodemographic factors (short version).

Covariate/Factor	Statistic Test	*p* Value
Gender	Pearson’s Chi-Square	0.470
Age	ANOVA	0.046
Education	Fisher’s Exact Test (Monte Carlo approach, IC 95%)	0.001
Barthel Index for Activities of Daily Living (score)	Pearson’s correlation coefficient	0.515
Lawton & Brody Instrumental Activities of Daily Living (score)	Kruskal–Wallis	0.222
Financial status	ANOVA	0.228
Caregiver transition	Kruskal–Wallis	0.291
Family/friends support	Mann–Whitney	0.091
Professional support	Student’s *t*	0.764
Private support	Student’s *t*	0.113
Knowledge about community resources	Student’s *t*	0.006
Demand for health services	Pearson’s Chi-Square	0.793
Demand for social services	Student’s *t*	0.691
Inclusion in caregiver’s support program	Fisher’s Exact Test (Monte Carlo approach, IC 95%)	0.103
Knowledge about the statute of the informal caregiver	Pearson’s Chi-Square	0.103
WHO Well-Being Index (score)	ANOVA/Pearson’s correlation coefficient	0.001

**Table 5:** Observed frequencies of education level by health literacy categories (valid N = 258).

Health Literacy Categories (HLS-EU-PT)	Education Level							
	Does not know how to read and write	Knows how to read and write	4 years (primary school)	6 years (junior school)	9 years (basic school)	12 years (high school)	Higher education degree	Other
Inadequate	2	0	7	0	1	2	3	0
Problematic	0	2	9	5	13	23	42	1
Sufficient	1	0	15	4	14	26	39	0
Excellent	0	1	2	1	4	7	34	0

## Data Availability

The data presented in this study are available on request from the corresponding author. The data are not publicly available due to privacy reasons.

## Appendix A. Sociodemographic Data of the Informal Caregiver

Sociodemographic data about informal caregiver is presented in Table A1 in a more detailed version.

**Table A1:** Sociodemographic data of the informal caregiver—extended version.

Variable	Dimension	*N*	%
Gender	Female	231	73%
	Male	84	27%
Marital status	Married	167	57%
	Single	61	21%
	Divorced/Separated	51	17%
	Widowed	16	5%
Education	Does not know how to read and write	4	1%
	Knows how to read and write	3	1%
	4 years (primary school)	37	13%
	6 years (junior school)	15	5%
	9 years (basic school)	37	13%
	12 years (high school)	67	23%
	Higher education degree	131	44%
	Other	1	0%
Profession	Armed forces	1	0%
	Representatives of the legislative offices and executive institutions, directors and executive managers	26	9%
	Scientific and intellectual activities specialists	85	29%
	Technicians and associate professions	31	11%
	Administrative personnel	71	24%
	Personal and security services and sellers	45	15%
	Farmers and skilled agricultural, fishing and forestry workers	2	1%
	Skilled workers in industry, construction and craftsmen	13	4%
	Facility workers and machine operators	12	1%
	Non-skilled workers	16	6%
Financial status (average monthly income)	<EUR 665	81	28%
	EUR 665—1270	113	38%
	EUR 1270—1905	52	18%
	EUR 1905—2540	26	9%
	EUR 2540—3175	12	4%
	>EUR 3175	10	3%

## Appendix B. Bivariate Statistics—Health Literacy and Sociodemographic Factors

**Table A2:** Bivariate statistics—health literacy and sociodemographic factors (full version).

Covariate/Factor	Dependent Variable	Test	Significance
Gender	Health literacy—HLS-EU-PT categories	Pearson’s Chi-Square	*p* = 0.487
	Health literacy—HLS-EU-PT score	Student’s *t* Mann–Whitney	*p* = 0.618 *p* = 0.470
Age	Health literacy—HLS-EU-PT categories	ANOVA Kruskal–Wallis	*p* = 0.046 *p* = 0.049
	Health literacy—HLS-EU-PT score	Pearson’s correlation coefficient	*p* = 0.069
Education	Health literacy—HLS-EU-PT categories	Fisher’s Exact Test (Monte Carlo approach, IC 95%)	*p* = 0.001
	Health literacy—HLS-EU-PT score	Kruskal–Wallis	*p* = 0.274
Barthel Index for Activities of Daily Living (score)	Health literacy—HLS-EU-PT categories	ANOVA Kruskal–Wallis	*p* = 0.531 *p* = 0.560
	Health literacy—HLS-EU-PT score	Pearson’s correlation coefficient	*p* = 0.515
Lawton & Brody Instrumental Activities of Daily Living (score)	Health literacy—HLS-EU-PT categories	ANOVA Kruskal–Wallis	*p* = 0.380 *p* = 0.222
	Health literacy—HLS-EU-PT score	Pearson’s correlation coefficient	*p* = 0.227
Financial status	Health literacy—HLS-EU-PT categories	Fisher’s Exact Test (Monte Carlo approach, IC 95%)	*p* = 0.409
	Health literacy—HLS-EU-PT score	ANOVA Kruskal–Wallis	*p* = 0.228 *p* = 0.287
Caregiver transition	Health literacy—HLS-EU-PT categories	Fisher’s Exact Test (Monte Carlo approach, IC 95%)	*p* = 0.375
	Health literacy—HLS-EU-PT score	ANOVA Kruskal–Wallis	*p* = 0.476 *p* = 0.291
Family / friends support	Health literacy—HLS-EU-PT categories	Pearson’s Chi-Square	*p* = 0.557
	Health literacy—HLS-EU-PT score	Student’s *t* Kruskal–Wallis	*p* = 0.173 *p* = 0.091
Professional support	Health literacy—HLS-EU-PT categories	Pearson’s Chi-Square	*p* = 0.967
	Health literacy—HLS-EU-PT score	Student’s *t* Mann–Whitney	*p* = 0.764 *p* = 0.856
Private support	Health literacy—HLS-EU-PT categories	Pearson’s Chi-Square	*p* = 0.305
	Health literacy—HLS-EU-PT score	Student’s *t* Mann–Whitney	*p* = 0.113 *p* = 0.284
Knowledge about community resources	Health literacy—HLS-EU-PT categories	Pearson’s Chi-Square	*p* = 0.177
	Health literacy—HLS-EU-PT score	Student’s *t* Mann–Whitney	*p* = 0.006 *p* = 0.007
Demand for health services	Health literacy—HLS-EU-PT categories	Pearson’s Chi-Square	*p* = 0.793
	Health literacy—HLS-EU-PT score	Student’s *t* Mann–Whitney	*p* = 0.910 *p* = 0.856
Demand for social services	Health literacy—HLS-EU-PT categories	Pearson’s Chi-Square	*p* = 0.779
	Health literacy—HLS-EU-PT score	Student’s *t* Mann–Whitney	*p* = 0.691 *p* = 0.899
Inclusion in caregiver’s support program	Health literacy—HLS-EU-PT categories	Fisher’s Exact Test (Monte Carlo approach, IC 95%)	*p* = 0.103
	Health literacy—HLS-EU-PT score	ANOVA Kruskal–Wallis	*p* = 0.755 *p* = 0.679
Knowledge about the statute of the informal caregiver	Health literacy—HLS-EU-PT categories	Pearson’s Chi-Square	*p* = 0.103
	Health literacy—HLS-EU-PT score	Student’s *t* Mann–Whitney	*p* = 0.672 *p* = 0.793
WHO Well-Being Index (score)	Health literacy—HLS-EU-PT categories	ANOVA Kruskal–Wallis	*p* = 0.001 *p* = 0.001
	Health literacy—HLS-EU-PT score	Pearson’s correlation coefficient	*p* = 0.001
